# Supplementary figures and images for: The fitness of an introgressing haplotype changes over the course of divergence and depends on its size and genomic location
Source: PLoS Biol. 2023 Jul 17;21(7):e3002185. doi: 10.1371/journal.pbio.3002185 (PMC10374083; doi:10.1371/journal.pbio.3002185)

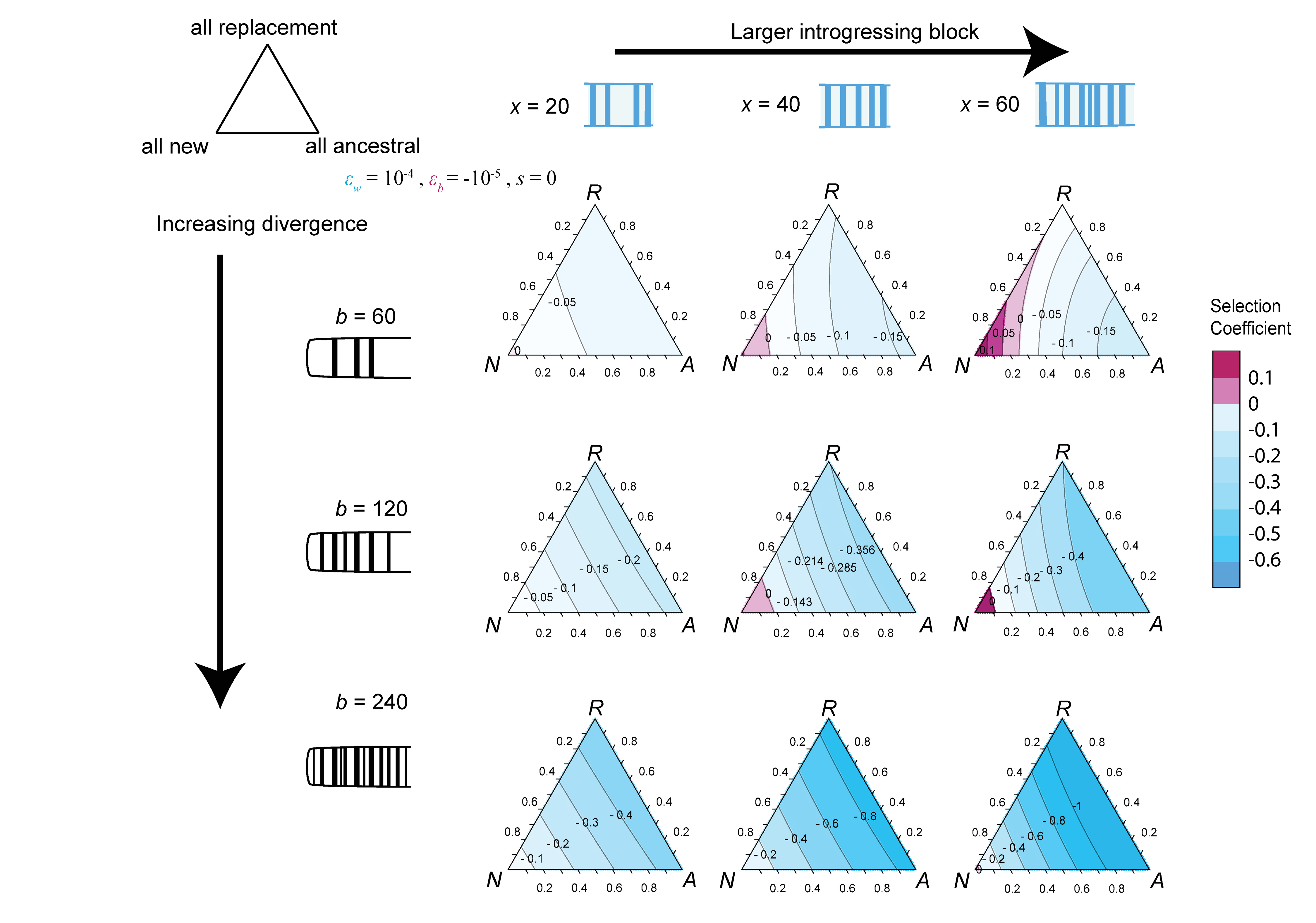

Supplement: S3 Fig — Examining how the selection coefficient on an introgressing haplotype changes as the fraction of substitutions it carries varies between new, ancestral, and replacement alleles, as in Fig 3 but using approximation in Eq (5). The code underlying this figure can be located in S1 File. (TIF) [file pbio.3002185.s003.tif]
