# Supplementary material for: The fitness of an introgressing haplotype changes over the course of divergence and depends on its size and genomic location
Source: PLoS Biol. 2023 Jul 17;21(7):e3002185. doi: 10.1371/journal.pbio.3002185 (PMC10374083; doi:10.1371/journal.pbio.3002185)
Supplement: Attachment — Submitted filename: ResponseToReviewers.docx [file pbio.3002185.s008.docx]

Response to Reviewers (Reviewer comments in red and tabbed).

Editor:

IMPORTANT: During the cross-commenting between the reviewers, and discussion with the Academic Editor, several issues emerged. One is that you do not refer to the substantial theory on introgression with selection and recombination (only citing Barton & Bengtsson 1986 in passing); a block of genome with a net selective advantage that is greater than recombination will increase, and this leads to predictions about the lengths of introgressing blocks (Sachdeva & Barton 2018). You would need to consider how this effect would contribute. Another concern is that you have not properly accounted for interactions with loci across the genome, rather than within the block - this could invalidate your conclusions.

We have addressed both of these concerns to the best of our ability, both by expanding our introduction and discussion of prior literature and integration of the Sachdeva & Barton papers, and some considerations on how the non-linear relationship between block fitness and block fixation probability demonstrated in Sachdeva and Barton impacts our results. Finally, we have clarified within our model assumptions and the text that we do account for interactions with loci across the genome, and that indeed these interactions drive the qualitative change in fitness of introgressing blocks early vs late in divergence.

Regarding the idea of a simulation (raised by reviewer #1), the Academic Editor thought that while this could be helpful as an illustration, a serious study would take a substantial amount of work to do properly. For example, if the simulation did not include recombination, it would merely replicate your analysis, whereas if it did, one would need to simulate over an extensive range of parameters, and also extend the analysis to include recombination. That would be a different paper, perhaps for the future.

We appreciate the editor’s understanding of the difficulty of generating such simulations. We do agree with both Reviewers 1 and 2 that incorporating recombination, even in a simulation framework is an important next step and is high on our priority list.

**Reviewer 1**

This could easily be misunderstanding on my part, but I'm struggling to understand the distinction between introgressing ancestral vs. derived variation with respects to the predicted effects on fitness. More specifically, the prediction that introgression of ancestral variation should be more deleterious seems like the opposite of what I'd intuitively expect. If A is donating ancestral alleles to B, presumably those alleles were also present in the common ancestor of A and B, which means they've been tested on at least part of B's genetic background at some point in the past. For this reason I'd expect the introgression of ancestral alleles to be less disruptive of epistasis, so the assumption that introgression of ancestral alleles removes b-1 epistatic effects seems a bit extreme? I'm also confused by the assumption that ancestral alleles "remove a direct fitness effect", as it seems to imply that ancestral alleles are exclusively selectively neutral and derived alleles always have an effect on fitness? Again, I'm not sure if there's any problem with the model construction or if some clarification is just needed in the text (particularly under the "selection on individual alleles" section).

We have re-written the presentation of the model. Crucially, all of the fitness effects are being compared to the ancestor. In that light, the ancestral allele has no fitness effects. Instead, it is replacing the fitness effects of whatever the diverged allele fixed in the recipient population is. E.g. – it will remove epistasis between that diverged allele and other diverged alleles as well as its selective effect. We hope that the revised manuscript makes this assumption of the model much clearer.

My other major suggestion would be to do a small forward simulation study to test the model predictions, especially one that incorporates the effects of recombination. I don't consider it necessary if it's too difficult and/or time-consuming, but I think it would really tie things together.

This is a great suggestion, but computationally turns out to be a bit more complicated, as we’d be required to make some assumptions about the linkage map in general, and between the diverged alleles specifically. As many different sets of linkage parameters would likely need to be tested, we reason it’s outside the scope of this manuscript and have chosen to leave this to a future study.

**Reviewer 2:**

Dagilis & Matute use a very simple model of introgression, to help understand the relation between recombination rate and size of introgressing block. This is timely, and relates directly to data. However, I think that the model is rather too simple, and needs to be strengthened if this paper is to be appropriate for PLoS Biology.

- The model assumptions need to be explained more clearly at the outset - they do gradually emerge, but quite late. Introgressing blocks are treated as non-recombining units, and the model focusses on their marginal advantage. Recombination is not included, which makes it hard to relate to the primary observation that they discuss - the relation between block length and recombination rate.

We have re-structured the introduction of the model so that the exact assumptions are made clear from the outset. While we do not consider recombination explicitly, our model also presents several predictions regarding the genomic location and direction of introgression.

- The focus is on (pairwise) epistatic interactions. However, even with no epistasis, there is an effect of block size: establishment probability and rate of increase are proportional to the net selection on the block. Thus, larger blocks necessarily increase faster. Indeed, even with recombination, a block can increase intact provided net selection is faster than recombination. This leads to an advantage for large blocks if selection is proportional to block length, and to moderate sized blocks if selection increases with sqrt(length) as it would if introgressing alleles have a distribution of effects) (Sachdeva & Barton, 2018 Genetics). These results are clearly relevant to understanding the relation between block size and recombination rate.

We appreciate being pointed to Sachdeva & Barton, somehow those papers had escaped our attention. They both are highly relevant to the content presented here. We have highlighted their relevance in both the introduction and discussion of our results.

- Having said that, in a large population and with high recombination rates, it is ultimately the selection on individual alleles that determines whether they can invade. That makes D&M's analysis somewhat relevant - but it is hard to see that recombination, and hitch-hiking, can be ignored when one considers blocks with multiple loci. There is a well-developed theory for rates of introgression with selection (including epistasis) and recombination, and it seems odd to avoid this.

While there is certainly a rich literature considering introgression of neutral alleles in association with deleterious alleles of various types, we aimed to ask what the fitness of those individual alleles would be over the course of divergence, and how the total effect of a block would then change. These are in many ways complimentary approaches, and we are not aiming to re-generate known results.

In the main text, additive and epistatic coefficients are assumed the same. This seems unnecessary, since in reality coefficients vary widely, and one can get expressions involving the mean and variance of coefficients. This will be easiest if one works with log fitness and then sums over loci (and pairs of loci) - which anyway seems more natural.

While we assumed the effects were the same for Figure 2, our analytical results and conclusions definitely do not rely on that assumption. To clarify, we now show a Figure 2 in which direct effects are an order of magnitude stronger, and have included Supplementary Figure 1 which explores a broader still set of epistatic parameters. While expressions including the mean and variance of the selection coefficients are possible, they depend highly on both the evolutionary trajectory of both populations and the structure of the epistatic interaction network. We now clarify that we are dealing with the mean effects specifically, and are assuming that variance is low.

Surely there are far more epistatic interactions with substitutions across the genome than with those in the homologous block? So, surely the relative sizes of the whole genome vs the block should enter? I worry that something crucial has been missed here. Along the same lines, on line 68 it's suggested that positive epistasis between alleles within a block will increase their fitness. Yet surely, such interactions across the whole genome will have a much larger effect??

We clearly failed in our writing, as our model explicitly counts such interactions (and indeed, these interactions with the rest of the genome drive qualitative differences between introgression into a recently diverged vs highly diverged population). To fix this, we have re-written large sections of both the model and results section and clarified Figures in any way we could think of. To clarify further - the parameter *b* is the number of derived alleles in the recipient genome, not simply on the block. Thus, as divergence increases (and so does *b*), the fitness of introgressing alleles rapidly changes. The relative size of the block vs divergence is therefore just x/b, and indeed the number of interactions across the whole genome eventually swamps any potential co-adapted interactions among the introgressing alleles.

One can easily use this framework to understand the "sieve" of selection: if one assumes a random distribution of coefficients, then within populations these will be established at a rate proportional to their selective advantage. Such an analysis is presumably somewhere in the "adaptive walk" literature.

To our knowledge, no analysis that is equivalent to what we present here exists. Part of the issue is that while epistatic effects may be in some random distribution at the start of divergence, as populations evolve they move into different areas of the fitness landscape and later draws from the distribution are not independent of prior ones – the assumed structure of the fitness landscape is therefore crucial to the resulting coefficients. This is in part why we present the marginal fitness of an introgressing block of alleles given some epistatic parameters – the actual value of those parameters is liable to change given a variety of evolutionary scenarios. We now address this in the results and discussion.

30 Pialek and Barton (Genetics 1997) might be a better reference for adaptive introgression than Barton (2001)

Corrected

47-75 and elsewhere - The writing is sometimes baffling. For example, the term " a negative relationship" is often used; I found that it took some thought to work out what this meant. More direct language would help (eg blocks in low-recombination regions tend to be larger"). Lines 384 I also found confusing.

We have re-written many of these sections.

In Fig 1A, the bars have different widths, which suggests introgression of blocks of varying size. But, aren;t they actually representing substitutions at individual loci?

The width differences are mostly illusory, as different bars sometimes overlap each other. We have attempted to make this effect less pronounced by more equally spacing the blocks.

228 Why should between population epistasis be several orders of magnitude larger than within-population epistasis?? I don't think that emerges from a "Sieve"model.

This observation emerges from our prior simulations of adaptation based on a yeast gene knockout epistatic interaction network. We expand our discussion of why this pattern might be observed more generally – with within population interactions being exposed to selection while between are quite close to a random sample among all possible interactions. We have, however, softened our language around this being the expectation, and now consider a wider parameter range.

**Reviewer 3:**

1. Comments on the model

● It would be helpful to clarify what the "small haplotype" (L80 and L437) considered throughout the manuscript exactly represents and what it involves for the model (is it small enough that recombination is unlikely?). The term is a bit confusing because an analysis of the size of the introgressing haplotype ("x") is carried out where large versus small haplotypes are considered (e.g. L85).

These terms are purposefully vague as the size of small vs large depends on several parameters in the model which, to our best knowledge, are unknown in real data-sets. In particular, both of the epistatic parameters in our model play a large effect, but have only really been studied in a single system (yeast knockout mutations) and we are cautious about extending the conclusions derived from those particular parameters to the field more broadly. We now clarify that the inflection point (and therefore small vs large) will vary by system and in particular by epistatic network.

Note that because the inflection point may change with total divergence, even equation 7 is not a complete accounting of what "small" and "large" mean, and this in part depends on the types of substitutions being considered. If all substitutions are expected to have really weak effects (both direct and indirect), small might refer to 1000s of substitutions on a single haplotype. On the other hand, small refers to only a couple substitutions of large effects (Figure 2).

● The model also assumes that each derived allele interacts with all other derived alleles fixed in the population leading to the combinatorial term in equation 1 (L109). This way of modelling should be better grounded in biological considerations. If one thinks about genetic networks, it seems more realistic that gene interactions occur within the same network and not between different networks. In that case, a derived allele will interact with only a subset of the total substitutions, making the contribution of Ew to the fitness of introgressing haplotypes weaker than in the present work. Moreover, genetic networks are highly structured, so even within a given network, only mutations affecting "hub genes" will likely interact with all other mutations in the network. In contrast, mutations in "peripheral genes" may interact with a limited number of mutations.

Yes - network structure is likely to impact the actual frequency with which alleles interact with each other. However, this will primarily affect the variance of the expected fitness effect, while the expected value will still be reflected by the model we describe (see, for instance Livingstone et al 2013 for an example in how network structure can largely be averaged). On the other hand, if substitutions are also biased in respect to network structure (for instance, hub genes less likely to evolve), then the variance in epistatic effects becomes rather large, and our approximations far less accurate. We add these considerations to the methods section.

● The parametrization of the model should be better justified. In particular, in Figs 2 & 3 Eb is an order of magnitude weaker than Ew. This setting amplifies the effect of Ew relative to Eb, as demonstrated in the analysis of the inflection point (L232). The authors mention that simulations confirmed this setting in Dagilis et al. 2019 (L137, L149), but more details on the rationale of this choice should be given in the present paper.

We have added further discussion of why these particular parameter values where used for illustrative purposes, and modified language throughout to make sure we discuss what the relationships in equation 7, for instance, suggest in general, no matter the choice of parameters. Additionally, we have added a supplementary figure exploring a broader range of epistatic parameters.

An interesting point made by Dagilis et al. 2019 is that Ew should get stronger with divergence because the fate of a new mutation primarily depends on its direct effect at low divergence, while it primarily depends on how well it interacts with other substitutions at higher divergence. On the contrary, Eb should change little with divergence because those interactions are not tested by selection (in allopatry). Consequently, the ratio of Eb to Ew varies with divergence, so the inflection point also does (equation 7 on L225). If drift dominates population divergence, then we can expect Eb to be similar to Ew regardless of the divergence, so Ew's contribution would be less significant for the fitness of an introgressing haplotype. These are important points that could be mentioned in the present manuscript.

It's true that both e_b_ and e_w_ will vary over the course of divergence (as in all likelihood will *s*). We now address this somewhat in the discussion. However, as the actual dynamics of these parameters depend highly on the actual evolutionary scenario (divergent selection vs local adaptation vs drift vs severe inbreeding), we chose to represent the fitness of introgressing alleles in general, and allow our readers to consider what values of ew and be might be reasonable for their organism of interest at the level of divergence they observe.

Similarly, on L159 it is stated that "epistasis may often be orders of magnitude weaker than direct fitness effects". Could you please justify your statement? If this is generally true, why then setting "s=Ew" in Fig 2?

There was no particularly good motivation for the *s* chosen in the previous version of Figure 2 (and indeed this choice was confusing for some reviewers). We have in turn changed it so that *s* is an order of magnitude larger than ew.

● "Selection on haplotypes" (L167): as it stands, the authors give the approximation of the effective selection on an introgressing haplotype when the three types of alleles contribute (eq. 5). Next, the equation ignoring the contribution of replacement alleles (eq. 6) is provided. But the equation only considering the contribution of newly derived alleles is missing. Could you please add this after L187 equation (i.e. when f=0)? Even if this is straightforward, it helps a lot in understanding the results of Fig 2A, and the relative contribution of Ew (quadratic dependence on "x") versus Eb (linear dependence on "x" and "b").

We have added this equation.

● "Selection in diploids" (L250): I would suggest moving these important results (Fig. S3) to the main text. Reminding that introgressed alleles are rare would help us understand that the model uses only two terms (a1 and a2) for epistasis. Finally, the effect of direct effects' dominance ("h") is not commented on anywhere. Could you please briefly describe the impact of "h" on your results?

We appreciate that the reviewer was interested in the diploid results as well. We have decided not to move them into the main results, for several reasons. First, as we note in the paper, the actual dominance of epistasis is generally unknown. Second, while there are large quantitative differences in the model predictions based on dominance, the qualitative differences are much smaller. That is, our main points about potential ease of introgression early in divergence by large blocks remain largely unchanged, and at later divergences we still expect a largely negative relationship between block size and fitness. We have added an additional set of figures exploring the effects of *h* and comment on these in the text.

I appreciated that the authors tried to test their model predictions using a published dataset of D. melanogaster. However, as most model predictions have to do with the size of the introgressing haplotype (at early divergence, the introgression of large haplotypes should be favoured compared to small ones), it would be more suitable to use a dataset where these sizes are reported. As mentioned in Duranton & Pool 2022, it seems that ancestry tract lengths are available for the North American D. melanogaster dataset (admixture of European and African populations, Corbett-Detig & Nielsen 2017). Otherwise, several such datasets exist between more or less diverged species (e.g. drosophila flies: Turissini and Matute 2017, coccidioides fungi: Maxwell et al. 2019, honey bees: Calfee et al. 2020 and Nelson et al. 2016, european sea bass: Duranton et al. 2018).

We would have loved to include more species sets in our comparisons, but the differences in the types of data made comparative results fairly difficult. In particular, we wanted to focus on relative branch lengths in each of the diverged windows, and we could not come up with a simple transformation of prior data to achieve this (specifically for the Corbett-Detig & Nielsen dataset).

● I liked that the authors tested the effect of divergence, measured with PBS, on the fate of introgressing haplotypes. In Fig 4D, the authors observe that the windows that diverged the less in the host population (OOA2) are also the ones that are introgressed the most, which agrees with the model predictions. However, two observations are not congruent with the predictions.

First, the OOA2 population has diverged more strongly than the West population, but the direction of introgression goes from West (source) to OOA2 (host), which is contrary to the prediction that the less diverged population should more easily be introgressed (L495). Could you explain this discrepancy?

We now discuss this in the paper, but introgression direction has been determined more by the direction of migration than any selective differences. The migration in question here is the Triangle trade, with a cycle of Africa -> North America -> Europe -> Africa. This makes the introgression from North America into Africa unlikely.

Second, the windows that diverged the more in the source population (West) are the ones that introgressed less (Fig S4C), which is contrary to the predictions that these diverged windows should bring new co-adapted alleles to the host population (L354). Could you explain this discrepancy?

This was difficult to display in our previous versions of the figures – it is true that we do not see support for higher West branches leading to higher introgression. But this pattern is largely driven by the overall low West branches, with very little power to discern any patterns. Notably, there is no significant relationship between the West branch and fD in a linear model, which we now show in Figure 4.

The authors indicate an over-representation of windows in which the West population shows rapid evolution among the introgressed regions (L342, L413). Still, I think that evidence is limited based on Fig 4B. Instead, Fig S4C shows the opposite trend.

We have corrected the discussion around these points. We based this statement on a fairly arbitrary cut-off of f_d>0.5 counting as “high” introgression demonstrating the following pattern:

But further evaluation of the data found no real link between f_d and the West Branch in our data, with a potential negative link in a joint linear model (or when including negative PBS values).

I think these patterns are not so easy to interpret because, ideally, one would like to know the divergence state of the windows in the source and host populations. Would it be possible to make a plot where PBS in the West is represented on the x-axis, PBS in OOA2 on the y-axis and colours indicate the introgression rate, fD?

The ternary plot with fD values was aiming to do this, as we use normalized branch scores and the sum of all three scores is always 1. In trying to answer this reviewer comment we realized that the density of points really was not coming across in the current figures. For instance, the plot the reviewer asked for looks like this:

Where the marginal histograms show that the vast majority of the data lies clustered, making it quite difficult to discern any patterns regarding f_d. We now include the West branch in figure 4, and show density of points rather than color by recombination or branch score, and hope that this makes the relationships easier to interpret.

● I believe the presentation of the results could be improved, and more details should be given for a reader unfamiliar with the biological system.

We have rewritten many sections of the paper to further clarify and better present our results.

Fig S4:

- Legend "Inclusion of negative fD values": Please, explain what negative fD values mean (introgression from OOA2 toward West?).

We now explain that negative values indicate introgression between a different pair of species (West and OOA1).

- The meaning of the colour scale is missing.

We have changed this figure heavily. In particular, we no longer use negative fD values, as interpreting these (which indicate introgression in an unexpected direction) is extremely difficult, and in general it is advised to only interpret fD in the plausible direction of introgression.

3. Comments on the Introduction

I believe the introduction does a good job of presenting the state of the art. However, it is also quite compact, with complex concepts addressed. As a result, it could be hard to follow for non-expert readers. Below are some suggestions:

- L57 - 59: It is confusing to first state the correlation in terms of the "size of the introgressing haplotype" to explain the negative correlation with the introgression rate observed in swordtail fish, butterflies, etc. And then, state the correlation in terms of "the recombination rate" to explain the negative correlation observed in D. melanogaster. That way, the fact that the relationship is reversed in D. melanogaster does not appear straight away (as the recombination rate and the size of the introgressing haplotype are negatively correlated).

This paragraph seems to have been a headache for multiple reviewers and several subsequent editors, so we have changed it substantially. Hopefully the writing is more clear now.

- L63-64: "heterotic effects resulting from overdominance" is not easy to understand. Please explain in a couple of sentences what you mean by that.

We have re-written much of this paragraph.

4. Minor comments

- L102: "direct and digenic epistatic fitness effects": Could you please add "(individual)" after "direct", and use "pairwise" instead of digenic (as it is used a single time).

Fixed

- L133: a "s" is missing in "as well a[s] b".

Fixed

- L220: Please, note that for the inflection point analyses, the replacement alleles are ignored.

Fixed -

L262: Please, clarify what "a1 and a2 are roughly proportional" means.

Fixed

- L279: "recombination free approach": The formulation is a bit confusing.

We have reworded this

- L284 - 287: The explanation is unclear to me. Could you please reformulate it?

We have further expanded this explanation.

- L305: "This introgression event represents an opportunity to test some of the predictions of our model" and L309: "Here, we use that data to test several predictions of our model" is repetitive.

Fixed

- L469: The conclusion is a bit long. I think the text between L480 and L515 could be shortened as most of it is already discussed elsewhere in the manuscript.

We have largely removed this section

- L531 - 539: Please, define population A as the source, and population B as the host.

We now term these source and recipient.

- L548: there is a typo in "XA and XA" → "XA and XB".

Fixed

- Fig 1:

Panel C: Add Lost interactions "in population B".

Panel C: Indicate the number of substitutions "b" and the length of the introgressing haplotype "x" on the cartoon.

Legend: Please, explain the symbols used (b, s, Eb, Ew), and the significance of the bars (black, blue, dashed).

Fixed

- Fig 2:

Title: it could be renamed with a more specific title like "Results for a haploid model".

Fixed.

Legend:

"between each other" → "between introgressed derived alleles".

Fixed.

"(dashed line)": this is a typo, only direct effects are not represented with a dashed line but a linear solid line.

Fixed.

"When the introgressing haplotype is only carrying novel alleles": please, add "(f=0)".

Fixed.

"with no replacement alleles": please, add "(xR=0)".

Fixed.

- Fig S2: Please, explain the meaning of a1 and a2 in the legend.

Added.

- Fig S3: What value of "h" was used in these analyses? Moreover, there is an error in the legend: the "fraction of the haplotype that is replacement alleles (f)" should be the "fraction of the haplotype that is ancestral alleles (f)".

*h* was kept at ½ for these figures, but we now add a row of figures modifying *h* (but not a1,a2).
