# Supplementary material for: The fitness of an introgressing haplotype changes over the course of divergence and depends on its size and genomic location
Source: PLoS Biol. 2023 Jul 17;21(7):e3002185. doi: 10.1371/journal.pbio.3002185 (PMC10374083; doi:10.1371/journal.pbio.3002185)
Supplement: Attachment — Submitted filename: ResponseToReviewers_2.docx [file pbio.3002185.s010.docx]

Editor:

a) Please address the remaining requests from reviewers #2 and #3.

We have incorporated all comments from both reviewers, except for one equation from Nick Barton which sadly was lost in formatting. If you would like us to include this equation, we can reach out to Nick and ask for the actual equation he was suggesting we include.

b) Is there any way of making the Title more informative? I quite like the almost philosophical tone of your current one, but it might be helpful to include the main findings (I couldn't think of a succinct way of doing this...).

We have debated a little bit on this, but because our results are fairly broad we’re not sure what a great title that includes the results would be. “The fitness of an introgressing haplotype changes over the course of divergence and depends on its size and genomic location” ends up being quite long, but might work.

c) Please address my Data Policy requests below; specifically, we need you to supply the numerical values underlying Figs 2AB, 3, 4BC, S1, S2, S3, S5ABCD, S6AB (and/or the code required to generate them), either as a supplementary data file or as a permanent DOI’d deposition.

We now include the Mathematica script used to generate the illustrations of the equations as well as the R script to generate figures concerning the melanogaster analysis in a zipped archive as File S7, and we have uploaded these scripts and data to github.

d) Please cite the location of the data/code clearly in all relevant main and supplementary Figure legends, e.g. “The data underlying this Figure can be found in S1 Data” or “The data underlying this Figure can be found in <https://doi.org/10.5281/zenodo.XXXXX>” <https://osf.io/XXXXX>

We have done so.

e) I note that you mention two of the reviewers (Barton, Fraisse) in the Acknowledgements. While we appreciate the sentiment, this is against PLOS policy, so please could you remove this?

We now thank our reviewers in general without naming them.

Reviewer #1:

The authors have made extensive revisions to the manuscript text in response to a thorough set of reviewer comments. I believe these changes have greatly improved the manuscript's clarity, and in retrospect, the simulation study was probably an unreasonable ask. I have no further comments or concerns.

We thank the reviewer for the positive feedback.

Reviewer #2:

[identifies himself as Nick Barton]

This revision is a considerable improvement, and most responses are reasonable. However, some sections are still confusing, and need to be fixed before publication.

124 There are several problems with the very first paragraph in this section.  A "haplotype" is never defined, and it is not stated that recombination will be ignored.  It needs to be said right at the start that we consider a small non-recombining block, introgressing from a into b.

We have changed the wording here – ironically an earlier version of the ms had precisely that wording, but some friendly reviewers suggested haplotype is more clear. We use both terms now.

126 The # of substitutions in A, a, is never actually used.  Better to say at the start that all that matters are the effects of the alleles carried by the small block, which will interact with the b alleles in the recipient genome.

Fixed.

128 ""the marginal  fitness of a rare introgressing haplotype is (WI) compared to the average individual in the recipient population (̅ ), and calculate the effective selection coefficient () for the haplotype,  equal to WI/̅ -1" ([pdf](zotero://open-pdf/library/items/QHA4GQ2V?page=22))  What is meant here? Surely, we are comparing the marginal effect of the introgressing block with the marginal effect of the homologous block within the recipient. As it is written, we seem to be comparing the effects of a small block with the fitness of a whole individual - which is confusing.

We have adjusted the wording here – we are comparing the fitness of an individual carrying the haplotype vs one that is not.

The middle product term in Eq 1 needs to be written in the general form (with s_i, eps_w,i,j), as in Eq 8.  This can then be approximated by assuming an "average" s and eps_w.  Similarly in Eq 2: the general form should be stated clearly, and then approximated.

Fixed

132 "all fitness effects are multiplicative" cannot be literally true, since this is a model of epistasis!

We have reworded this – an alternative approach to fitness would be to assume that fitness is a sum, rather than a product, of the direct and epistatic effects.

147 "fitness depends.. at least quadratically on epistatic effects". No! The formula is linear in epsilon. What is meant is "quadratically on the number of pairwise epistatic interactions"

Fixed.

170, 195 and 247: the order of magnitude difference must depend on assumptions that went into the simulations; this will not generally hold.

We think we broadly agree in that this is likely a result of the particular epistatic network which was used for simulations (and we make statements to that effect on lines 244-246, , as well as including a broader exploration of parameters in Figure S1). In the current version, the parameters we use are both illustrative and our conclusions rely more heavily on the qualitative shape of the curves, or are generalized to any parameters when possible (e.g. Equation 8)

Eqs 5,6: There can be some further aproximation by noting that b>>1, and also that we expect b\*eps to be of the same order as s (or rather, this is a natural scaling to make the contributions to fitness comparable). We then expect Eq 6 to further simplify to: "(1 − 2)() − (_ − _b(1 − ))(b)"

Sadly, the formatting here seems to not have exported, so we are not able to further any derivations from Equation 6 here. In either case, we’re not sure we want to make the additional assumptions for an alternatively simplified Equation 6, as Equation 7 already is a simplified form.

222-224 I don't see that the quadratic term in x implies that the size of the haplotype has a larger effect than divergence, since b>>x surely?

b>>x only if *b* is quite large (e.g. we are later in divergence). But early in divergence, introgression may bring in numbers of alleles on a similar order to total divergence in the recipient population, especially if introgression is occurring from a highly diverged branch. We try to make clear in the text that this is the driver in different patterns expected in early vs late divergence.

Fig 3 - It is unreasonable to show such large values of x - which imply that the introgressing block is the same size as the whole genome (x~b).  This is perhaps less unreasonable for Drosophila, which has a weirdly small # of chromosomes, but  does not make much sense even there.

We disagree on this point – it’s certainly not the expectation for the vast majority of cases of introgression, but could be highly relevant for precisely the cases in which large blocks are not recombining – for example, horizontal gene transfer of a plasmid between bacterial strains, or introgression of inversions associated with pesticide resistance in mosquitoes. We agree that once x => b we are outside any reasonable parameter space.

456-457 Again, the quadratic vs linear comparison is misleading since one expects x<<b.

That is true, but we are considering specifically early divergence in which x may be on the same order as b, even if still smaller.

672 effect-> affected

Fixed.

Eq 9 should be written with explicit subscripts s_i and eps_w,i,j etc

Fixed.

Reviewer #3:

[identifies herself as Christelle Fraïsse]

The authors made efforts to address most of the concerns raised during the reviewing process. The updated version of the manuscript includes new results, such as in the Supp. Fig. 1 (exploring a broader range of within and between epistasis values), and Supp. Fig. 5 (showing additional results for a diploid model). The authors also elaborated on several points that needed to be clearer in the first version, such as the variation of epistasis parameter values with divergence and consideration of genes network. They are also more cautious now with interpreting the Drosophila data (and I liked the new version of Figure 4 with densities depicted). Overall, this work is of high quality and relevant to our understanding of introgression patterns. It provides exciting predictions to be tested in a comparative genomics framework. Therefore I fully support its publication, although I still have a few minor suggestions below. Note that line numbers refer to the "tracked changes"

version of the manuscript.

1) The authors do not consider the effect of recombination in their model in this updated version, which makes hard to draw conclusions on the relationship between recombination rate and introgression frequency. I understand that the authors failed to include recombination in their analytical model, and that running simulations would be cumbersome. They now briefly discuss how the effect of recombination may contribute to their model, which is OK. However, I'd suggest putting more emphasis on predictions about the direction and genomic location of introgression rather than on the relationship between recombination and introgression, especially in the abstract and Introduction.

We’ve modified the abstract to accommodate for the reviewer’s comments.

2) The new version of Fig. 2 uses a lower value of direct fitness effects (i.e. s=-10^-2 instead of -10^-3 previously). This change renders the parameter space in which the positive interactions can cancel out the deleterious effects of the introgressing block much reduced compared to the previous version. Basically, the selection coefficient of the block is positive only when divergence is low (b=30) and the haplotype is large (x>25), which would mean that the haplotype introgressing carries nearly all the divergent sites. Moreover, the selection coefficient of the large haplotypes is similar to that of short ones, while in the previous version, it was much higher. Thereby, the prediction that the introgression of large haplotype can be favored in early divergence is strongly dependent on the underlying fitness landscapes (i.e. direct and epistatic selection coefficients). This could be discussed a bit more along lines 267 - 283.

We have added a few sentences to highlight how these parameter values can change expectations in the early section of discussion, and expanded this point in the discussion. We do think, however, that if anything the figures we presented are a sort of “pessimistic” case for introgression, in which each allele is individually very deleterious. The fact that some introgression can be favored early in divergence (even if a large fraction of the genome must introgress) is in some ways reassuring. However, we have tempered some of the statements in the results in line with the reviewer’s comments.

3) Other minor points:

● L52 - 56: you list three patterns suggestive of the non-neutrality of introgressed regions. However, as it reads, it is unclear whether all patterns relate to humans or it is just the second one. Rephrasing would help to clarify.

We have adjusted these lines.

● L70: consider replacing "without recombination" with "in the absence of recombination".

Done

● L106: consider replacing "sex chromosomes" with "sex-limited chromosomes".

Done

● L121: consider replacing "location" with "genomic location".

Done.

● L383: typo in "...general case of positive within and negative between [ ] this leads..."

Fixed.

● L745: typo in "...the expected fitness should not be [effect]..."

Fixed.
